# Social Life of Females with Persistent COVID-19 Symptoms: A Qualitative Study

**DOI:** 10.3390/ijerph19159076

**Published:** 2022-07-26

**Authors:** Atefeh Aghaei, Ran Zhang, Slone Taylor, Cheuk-Chi Tam, Chih-Hsiang Yang, Xiaoming Li, Shan Qiao

**Affiliations:** 1 Department of Health Promotion Education and Behavior, Arnold School of Public Health, University of South Carolina, Columbia, SC 29208, USA; aaghaei@email.sc.edu (A.A.); rz1@email.sc.edu (R.Z.); ctam@mailbox.sc.edu (C.-C.T.); xiaoming@mailbox.sc.edu (X.L.); 2 South Carolina SmartState Center of Healthcare Quality, Arnold School of Public Health, University of South Carolina, Columbia, SC 29208, USA; 3 Department of Epidemiology and Biostatistics, Arnold School of Public Health, University of South Carolina, Columbia, SC 29208, USA; slonet@email.sc.edu; 4 Department of Exercise Science, Arnold School of Public Health, University of South Carolina, Columbia, SC 29208, USA; cy11@mailbox.sc.edu; 5 Technology Center to Promote Healthy Lifestyles, Arnold School of Public Health, University of South Carolina, Columbia, SC 29208, USA

**Keywords:** long COVID, female long haulers, persistent COVID-19 symptoms, social life, social role conflict, social stigma, social relationship, qualitative study

## Abstract

Persistent COVID-19 symptoms (long COVID) may bring challenges to long haulers’ social lives. Females may endure more profound impacts given their special social roles and existing structural inequality. This study explores the effects of long COVID on the social life of female long haulers. We conducted semi-structured interviews via Zoom between April and June 2021 with 15 female long haulers in the United States, purposely recruited from Facebook and Slack groups and organization websites related to long COVID. Interviews were audio-recorded and transcribed verbatim with consent. The interview data were managed using MAXQDA and examined by thematic analysis. Long COVID negatively affected female long haulers’ social lives by causing physical limitations, economic issues, altered social relationships, social roles’ conflicts, and social stigma. Long COVID prevented female long haulers’ recovery process. Physical limitations altered their perceptions on body, and family–work conflicts caused tremendous stress. They also experienced internalized stigma and job insecurities. This study provides insights into challenges that COVID-19 female long haulers could face in their return to normal social life, underscoring the vulnerability of females affected by long COVID due to significant alterations in their social lives. Shifting to new methods of communication, especially social media, diminished the adverse effects of long COVID (e.g., social isolation).

## 1. Introduction

The coronavirus disease 2019 (COVID-19) pandemic has resulted in 513 million infection cases globally with over 6.2 million deaths [1]. Although many people infected with COVID-19 have no or mild symptoms that last about two weeks, about 10–20% of individuals with coronavirus infection experience persisting symptoms even after recovering from the acute phase of the illness [2,3]. While the accurate number of people with long COVID is unknown, the Centers for Disease Control and Prevention (CDC) estimates that about 13.3% of people who had COVID-19 experience post-COVID symptoms at one month or longer and this proportion increases to 30% at 6 months after infection for patients who were hospitalized because of COVID-19. Based on estimates from UK’s Office for National Statistics, about 20% of people with COVID-19 have long-term symptoms beyond 5 weeks and this ratio decreases to 10% after 12 weeks [4]. Persistent symptoms related to COVID-19 can be multi-faceted. The most common symptoms are physical problems (e.g., fatigue and shortness of breath), while cognitive problems (e.g., difficulty concentrating) and emotional distress (e.g., depression) are prevalent as well [2]. According to a study in Italy [5], COVID-19 patients reported long-term problems such as fatigue (53.1%), shortness of breath (43.4%), joint pain (27.3%), and chest pain (21.7%) as well as reduced quality of life (44.1%). These post-acute sequelae of SARS-CoV-2 (PASC) or long-term COVID-19 symptoms are referred to colloquially as “post-COVID syndrome”, “long COVID”, or “long-term COVID” [6,7,8]. People with post-COVID syndrome are referred to as “long haulers” [9]. These persistent symptoms could cause health issues, thus significantly impacting people’s social lives (e.g., social relationships) [10].

Notably, females are disproportionately affected by long COVID. Although preliminary reports indicate that females have a reduced risk of severe disease and death from COVID-19 compared to males [11,12], the U.S. Department of Health and Human Services [13] reports that females comprise up to 80% of the patient population suffering from persistent symptoms [14]. This disproportional impact of long COVID on females could be attributed to specific social stressors that females face during the pandemic, including social isolation, unstable income, and the burden of caring for their families [11]. In addition to the psychological effects of social isolation on women such as depression and anxiety [15,16], social isolation also can influence their relationships with family and friends. Additionally, early studies have shown that females have been disproportionately affected by COVID-19-related unemployment and economic impacts compared to males [17,18], which could limit females’ ability to support themselves and their families [19]. As females play different social roles (i.e., wife, mother, primary caregiver, and employee), their vast responsibilities may lead to physical and psychological exhaustion, stress, and burnout in the context of the pandemic [20].

While many studies reported the physical symptoms of people with long COVID [4,21,22,23], other aspects of long COVID impact on people’s lives are overlooked. For example, Crook H et al. [21] reviewed 37 studies that reported the symptoms of long COVID. However, only 4 of these 37 studies [24,25,26,27] reported non-physical symptoms of long COVID (e.g., post-traumatic stress disorder, depression, anxiety, cognitive deficit, the requirement for personal care, and limited activities of daily living) [21]. Moreover, existing studies have focused more on the prevalence and types of symptoms rather than how these symptoms impact people’s lives [2,4,21]. Thus, there is a gap in knowledge about mechanisms that link long COVID symptoms to practical changes in the daily lives of people with long COVID. The higher impact of persistent COVID-19 symptoms on women’s social life [13,14] as a vulnerable group necessitates further research. However, there is a dearth of empirical studies among women who endure long COVID. To address these gaps, this study investigates the impact of long COVID on female long haulers by exploring the effects of long COVID physical and psychological symptoms on various aspects of women’s social life, such as their communication, social roles, perceived body image, and stigma. 

In this qualitative study, we propose a conceptual model that illustrates the impacts of long COVID on social aspects of female long haulers’ life. This model links the risk factors of long COVID symptoms such as body pain, physical limitation, stress, and fear to the social life outcomes such as social capital, conflicts in the treatment process and patient role, perceived body image, and stigma. By employing the conceptual model and analyzing the interview data, we scrutinized the impact of long COVID on female long haulers as a vulnerable group. 

## 2. Methods

### 2.1. Study Design

The current study was based on an online health promotion intervention among long haulers. The long haulers interviewed for this study were participants enrolled in the online health promotion intervention. Participants were recruited primarily using social media, with Facebook being the main social media platform through purposive and snowball sampling. Eligibility criteria included living in the United States, being 18 years of age or older, speaking and understanding English, having been infected with COVID-19, and having experienced at least one COVID-19 symptom lasting four weeks or longer after a COVID-19 diagnosis. There were 16 Facebook groups, 1 Slack group, and 2 organizations’ websites identified relating to these topics. The Facebook groups were identified using key search words such as “COVID-19”, “long hauler”, and “post COVID”. The organizations and their websites and Slack group were identified through the Facebook groups. All but three Facebook groups were private, and the Slack group was private, meaning only those who are members of the group have access to the content posted in the group. Upon receiving approval from administrators of the groups and organizations, posts including research recruitment flyer, study description, and contact information were shared in 8 Facebook groups, 1 Slack group, and 1 organization’s website. Seven of the Facebook groups, the Slack group, and the organization’s website were not geographically restricted; however, one Facebook group targeted residents of South Carolina. Recruitment took place over two and a half months from the end of March 2021 to mid-June 2021. In total, 17 participants completed a written informed consent form and participated in a semi-structured interview. This study was reviewed and approved by the University of South Carolina Institutional Review Board (Pro00109358). The semi-structured interviews were conducted online using the video teleconferencing software Zoom (Zoom Video Communications, Inc., San Jose, CA, USA) [28]. The interviews coincided with recruitment and took place from the beginning of April 2021 to the beginning of June 2021.

### 2.2. Data Collection

Semi-structured interviews were conducted using an interview guide (see Table 1). The interview guide covered the background of COVID-19 infection and related symptoms, the psychosocial impact of COVID-19, and social sources for support and resilience. Interviews were scheduled according to the availability of the interviewer and participants, and each interview was held one-on-one with only the interviewer and each participant present. After each interview, a 30 USD Amazon e-gift card was given to the participant as a token of appreciation. Data collection was performed until we reached data saturation confirmed by two researchers. After 13 interviews, we reached saturation and no new codes were identified from two remaining interview data. The total sample of 15 in our study was deemed sufficient as we reached saturation and sample size was larger than the minimum sample size (12 interviews) suggested for qualitative studies [29,30,31]. Each interview was recorded with verbal permission from the participant for transcription purposes. The transcription software Otter.ai [32] was used to transcribe the interviews, and all the transcripts were reviewed and edited by the interviewer. 

### 2.3. Data Analysis

The inductive approach for thematic analysis was applied in this study [31,33,34]. The thematic analysis consists of six stages: becoming familiar with data, developing initial codes, extracting themes, refining themes, labeling themes, and reporting [35]. The MAXQDA software [36] was used to analyze the interview transcripts. First, transcripts were reviewed multiple times for accuracy [37,38], and emergent themes (open coding) were identified [39]. Then, subthemes were categorized into five main themes (axial coding). In this thematic analysis process, initial codes were discussed during team meetings. The final codebook was obtained after reorganizing the codes as needed. This codebook contained definitions of the themes, exemplar quotes, and quote samples that did not fit into the categorization.

We then compared and contrasted themes to determine similarities, differences, and associations between findings. We employed standard and in-depth qualitative data analysis techniques during coding, including open coding, marginal remarks, axial coding, memo-writing, and comparisons. Peer debriefing and inter-coder agreement techniques were used to ensure the reliability of the analysis [40,41,42]. Inter-coder agreement reliability is a measurement of agreement between the coders about the similar data [43]. The codes and results were presented to two research team members who were not involved in the data analysis to discuss themes and related outcomes [40,42]. Verbatim quotes representing the themes were chosen to demonstrate the main findings.

## 3. Results

Fifteen participants qualified for the objectives of this study. Most participants were between the ages of 36 and 65 years (*n* = 12) (see Table 2). The primary distributions of participants by occupation were in the healthcare (*n* = 5), education (*n* = 4), and business field (*n* = 4). The geographic location of participants was concentrated in the eastern states (*n* = 10) which could have resulted from the convenient sampling in searching of the social media group of long COVID or could be a reflection of the high proportion of social media groups among long haulers in eastern states. 

**Figure 1:**
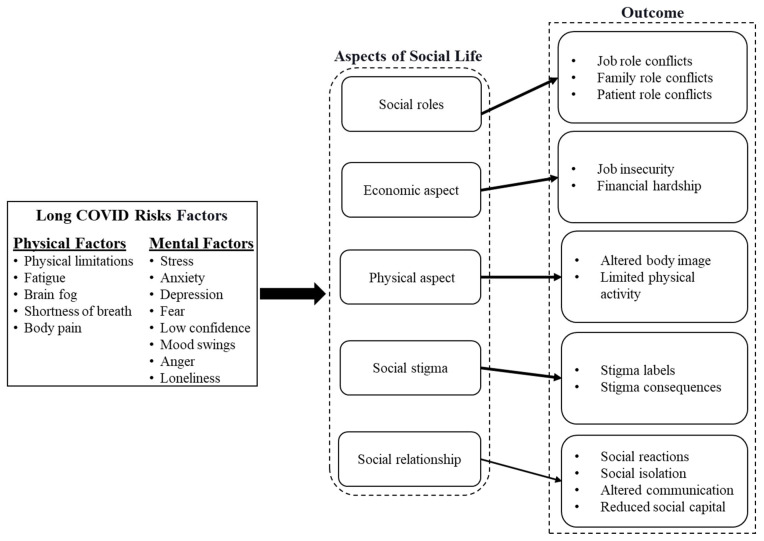
Different aspects of female long haulers’ social life affected by COVID-19.

Interviews with female long haulers identified five main categories: physical issues, economic issues, social relationships, conflict of social roles, and social stigma. The conceptual model (Figure 1) illustrates the links between impacts of persistent COVID-19 symptoms and their outcomes in female long haulers’ social life.

### 3.1. Physical Issues

One of the significant challenges in long haulers’ social life is their physical issues due to the persistent COVID-19 symptoms. These physical issues can be categorized into the “limited physical activities” and “altered perceived body image”.

*Limited physical activities.* Almost all the participants reported “declined mobility”, causing them to have “feelings of inactivity”. For instance, even performing the simplest tasks, such as “gardening or making a meal” led to negative feelings of being “like a couch potato”. Some participants reported that they had to “be cautious in doing physical activities” because they were still “in and out of doctors’ offices”. Almost all participants said they felt a “lack of energy” and quickly felt tired even after recovery. As a result, participating in social activities was difficult for them. One of them asserted, “I can go out with a friend, but I can’t do anything after that”. 

*Altered perceived body image.* The altered perceived body image is one of the characteristics reported by many female long haulers. These females had an erroneous perception of their bodies, believing that their current body condition is still functioning as it was before COVID-19. They could not accept their body’s new limitations due to long COVID symptoms. They even stated the impact of altered perceived body image on their recovery process and following through with treatments. Some participants reported feelings of “being tired of resuming the healing process”, because of their “weak and fragile body” and they experienced feelings such as “resentment” toward the spirometer because they “had to be still using it”. Thus, perceived “weak” body image caused them fear, anxiety, low self-confidence, and loneliness. More than half of the participants reported that they often “compared current physical abilities with pre-COVID-19 time” and “sighed for their current physical abilities”. One participant said, “this turned me into a homebody, which is anybody”. One participant said, “all my muscle is gone … mentally my body image had changed”. 

### 3.2. Economic Issues

The economic issue is another aspect of female long haulers’ social life affected by COVID-19. For most participants, “job insecurity” and “financial hardship” were the main economic issues that impeded their recovery. 

*Job insecurity.* Almost all the participants shared their concerns about their employment status. One participant reported the “fear of being unemployment” as even more stressful than the infection. Another participant said that a company “can fire you for absolutely no reason”. In this case, the participants were constantly looking for new jobs instead of focusing on their health improvement. One participant stated, “they just push my resume aside, so that’s been more of a stressor to me than the pandemic”. Two participants had to “quit their jobs to attend to family-related affairs”. Other participants noted that their “business is at risk” because of limitations in their ability to work caused by their persisting symptoms. One participant said, “because I have to cook… I have to breathe... so my business took a hit”. 

*Financial hardship.* The impact of the pandemic on the domestic as well as the global economy has affected some participants’ businesses, resulting in a “reduction in their income.” A participant explained that she was “forced to have a part-time job”. However, many participants had to “reduce their working hours” due to persistent symptoms. One participant described the pain she felt while working. These issues contributed to the participants’ unstable financial status, thus to the point that they “failed to pay medical bills”. Some participants mentioned that they were concerned about medical costs due to the possibility of losing their job. One participant was worried that the government would “stop paying COVID-19 claims”. This situation prompted some participants to “work overtime”. In these circumstances, nearly half of the participants experienced “interruptions in the recovery processes” due to financial issues or lack of “health insurance”.

### 3.3. Social Relationships

Persistent symptoms affected the social relationships of female long haulers. Based on interview results, this impact can be discussed in four themes: “social isolation”, “social reaction to long haulers’ symptoms”, “changed communication methods”, and “decreased social capital”.

*Social isolation.* All participants experienced social isolation due to changes and “restrictions in social relationships”. These challenges were rooted in their “limited energy” and weak physical condition, making social interactions difficult. Persistent “physical pain also impacted the maintenance and development of their social relationships”. One participant said, “if I’m on my feet too long... get pain... I haven’t been out with my friends”. Many participants reported that COVID-19 changed and “restricted their marital relationships”. Some participants who were infected with their spouses were afraid to approach each other due to symptoms. Some participants described that they should “end part of their social relationships to save energy for work activities”. One of them said, “if I go, then I’m probably gonna feel tired… I can’t risk that for my job”.

They also experienced social isolation due to the needs and requirement of COVID-19 prevention need. Several participants described the short time they spent with family and friends during the pandemic. One participant said, “the biggest thing that has impacted me personally is the loss of socialization”. In addition, some participants noted that staying at home and having limited relationships made them feel anxious and stressed. Some participants mentioned that kissing, hugging, and simply handshaking was done reluctantly to keep to manners. One participant said, “I was free from the hospital, and my daughter finally could hug me”. Several participants also mentioned some” physical and emotional conflicts” in dealing with social relationships. One participant said, “it’s been hard jumping back into that social life”. Additionally, another participant highlighted “a change in personality” by saying, “I feel like I’m not as extroverted or bubbly as I used to be”. 

*Social reactions to long haulers’ symptoms.* About half of the participants reported that some people, especially those who had never been infected, feel quite “doubtful about persistent symptoms”. One participant said, “some are incredulous that it’s happening, they are like, oh, just fight through it”. Other participants mentioned, “some medical professionals try to trivialize it or make their patients think that it perhaps might be in their head”. Moreover, the situation of long haulers is not understandable for some people. One of the participants asserted, “many people don’t understand; I could only last for about 30 min”. Several participants reported that “only people experienced COVID-19 could understand their feelings”, such as “the long COVID-19 Facebook groups because they are also going through it”. 

*Changed communication methods.* Most participants have experienced some forms of online communication. In this regard, some female long haulers said that they had already missed the opportunity to attend the public sphere. Still, through pandemic by the “emergence of new public spheres”, participants pointed out they “have taken benefit of different new applications and online forums” for social interaction culminated in the feeling of “belongingness”. One of the participants asserted, “I liked that because you are not alone”. Finally, as said by a participant about a long hauler advocacy group, “they’re trying to fight for even like disability rights for us”. 

*Decreased social capital.* In addition to the negative impact of social isolation on social capital, conflict in COVID-19 vaccination beliefs among family and friends caused a loss of social capital for a few female long haulers. Participants pinpointed that “reduced social support” and “limited social network” are adverse outcomes of decreasing social capital. One of the participants highlighted her higher level of vulnerability and said, “I’m a social person … but I’m more cautious now because of my symptoms, and I don’t like to be in crowds which I don’t know if people had their vaccine”. The other one mentioned, “I couldn’t really interact with people because there’s a lot of people that have not had their vaccine”. Consequently, less than half of the participants decided to terminate their relationship as one of them mentioned, “I’ve lost a lot of friends because of it, because… they’re anti-vaccine”. Another participant highlighted the roots of conflict in the beliefs to vaccination and said, “suddenly, people that have never had a political or social opinion, now do and so... that’s how it’s really affected me”.

### 3.4. Conflicts of Social Roles

Most participants experienced conflicts in their social roles that can be divided into three main themes: “conflicts related to job roles” and “conflicts related to family roles”, and “conflicts related to patient role”.

*Conflicts related to the job roles.* Participants’ job roles exposed them to different conflicts that revolve around their expectations and responsibilities in the workplace and their relationship with their employers. Some participants were affected by their “employer’s negligence”, which made them “feel helpless and ignored”. One of the participants said, “when we were sick… they didn’t call us one time. I noticed that they weren’t really there for us”. Some participants also reported not being permitted by their supervisors to leave the workplace when they had to or being unjustifiably pressured to work by their supervisors. 

In terms of “making a living”, participants turned out to have been struggling with financial problems intensified by COVID-19. Since their employers did not support them, they were pushed to “work overtime” or to “cover shifts when other staff members were infected”. One participant said, “when I was supposed to be on sick leave, I was in my bed, typing up stuff”. In addition, people who work under such conditions experience fatigue. As a result, their poor performance at work leads to “increased conflict with employers”. Especially for the head of the household, work role conflicts can cause them to wrestle with unemployment that was even more demanding and stressful than COVID-19 infection. Thus, some participants felt they had no choice but to push themselves to challenge themselves to return to business. One participant said, “I was trying to get to work and just struggling with that”. 

Some participants reported that “the pressure of work responsibilities” such as safe workplace requirements during COVID-19 was inevitable. The requirement to wear a mask to work was very difficult for participants with breathing problems. One participant stated, “professionally, that was the hardest thing, making sure that my students are safe”. This situation has become even more complicated for healthcare providers. As one nurse said, “I have never left my job because I have patients on my schedule”.

*Conflicts related to the family roles.* Most participants mentioned that they were expected to take care of the family even when they struggled with their persistent symptoms. This situation elevates the risk of a painful managing task for female long haulers. One participant said, “we both were positive… but I have to make sure my son is okay”. Furthermore, participants mentioned more expectations related to their unique role as mothers. One participant said, “my son was so stressed because I couldn’t take him to practice”. Additionally, “doing housework responsibilities” is another challenge for female long haulers. Almost all the participants reported that it is “difficult to handle daily house chores” in addition to work-related issues throughout the pandemic. One participant said, “even now, just cleaning my house sometimes is too much”.

*Conflicts related to patient role.* Female long haulers faced some conflicts between their patient role and their role as a COVID-19 survivor. As a patient, they need to have more rest and focus on their health but as the ones who passed the illness period and coming back to normal life, they were expected to fulfill their normal duties and tasks. This conflict caused some levels of misunderstanding between the female long haulers and their family, employer, and even friends. One of the participants mentioned “you don’t feel good, but you have to get to work, and then you have kids and family, and then, you know, you still push through even though you feel like crap... you are expected to do all”. 

### 3.5. Social Stigma

Experiencing social stigma had a negative impact on the social life of female long haulers. Two dimensions of social stigma, “stigma labels” and “stigma consequences”, were identified in this study. 

*Stigma labels.* All participants were labeled variously by other people. Two participants stated that they were identified as negligent to health principles, especially at the pandemic’s beginning. One of them acknowledged that people did not talk openly about being infected with COVID-19 due to fear of being stigmatized and blamed for “their careless behavior that caused the infection”. Another participant stated, “we had this sense of what did we do wrong”. Moreover, another two participants mentioned that they were identified as “danger to others” and labeled “risk factor”. One participant stated, “we felt like a leper”. Others explained that their “situation was unbelievable for some people” and triggered another type of stigma such as being “overdramatic” that was driven from a misapprehension of the COVID-19 deterioration. One of them noted, “a lot of my family members got it, but they didn’t get the after symptoms… they think I’m maybe being overdramatic”. Meanwhile, a few participants were labeled as “walking dead” people. One of them asserted, “there are so many stories… they collapsed, and after an autopsy, you know, it’s in their lungs”.

*Stigma outcomes.* Ultimately, stigmatization caused participants to “internalize the stigma”, “self-blaming”, and “fear of being judged”. Participants identified themselves through the labels perceived in their social lives. For example, one of the participants mentioned, “there’s a lot of question about why me? What did I do to have it?” and “you feel like you did something wrong”. Another one explained she “blamed herself for not being serious” about coronavirus prevention measures. Moreover, less than half of the female long haulers in this study restricted their social relationships because they avoided facing judgment and stigma. One of the participants asserted, “any symptom that I have… it’s embarrassing… I have to turn down like going with my husband’s family”. Other participants explained, “I feel like I have to keep proving to people that... I have stress of people getting believe it and not feel like I’m making this up”.

## 4. Discussion

This study makes a significant effort to explore the experiences of female long haulers and the impact of long COVID on their social life. In our study, many participants reported that their persistent COVID-19 symptoms hampered their physical activity and altered their perceived body image. Moreover, layoffs or increased household duties induced by the pandemic generated a slew of financial issues for them. Although almost all participants asserted profound changes in their social relationships, communicating with people who have similar experience on social media or social platforms allowed them to gain a sense of belonging and support. Our study also shows that social role conflicts are mostly related to the expectations of the surrounding people, and that female long haulers’ persistent symptoms prevent them from performing different social roles as competently as before. This situation ultimately leads to stigmatization of female long haulers, as well as their tendency to internalize the stigma, further impeding their return to a normal social life.

The main effect of persistent symptoms on female long haulers is a decrease in daily activity. Our findings show that participants easily become fatigued while engaging in physical activity, even during treatment. These findings are consistent with Shelley et al.’s study of individuals with persistent COVID-19 symptoms [44]. Participants in that study reported that COVID-19 had a profound impact on all aspects of their lives and made them inactive compared to their pre-COVID-19 status [44]. Moreover, another study found that persistent symptoms adversely affected people’s ability to engage in exercise, job duties, self-care, and housework, mostly by making them tired [45]. Many studies have pointed out that daily activities (e.g., housework and gardening) play an important role in improving the mental health and well-being of long haulers [46]; therefore, inactivity may have a detrimental influence on long haulers’ mental health. Moreover, some studies have mentioned that COVID-19 infections and symptoms have a negative impact on people’s perceived body image [47]. Our findings demonstrate that changes in the perceived body image among long haulers are rooted in their physical limitations caused by persistent symptoms.

In addition to the physical limitations caused by persistent symptoms on female long haulers, the COVID-19 pandemic has a huge impact on their employment and income, which further leads to economic losses [48]. In our study, many participants reported that long COVID directly or indirectly lead to unstable work status or job loss. Previous studies have demonstrated that many females working in the informal sector have limited access to benefits such as social security [49]. Large-scale unemployment could also have long-term effects on their economic independence and financial security [49]. The lack of jobs was particularly exacerbated during COVID-19, and females encountered additional barriers and challenges to re-entering the workforce. In fact, COVID-19 may worsen existing gender inequalities over time [50]. In our study, participants mentioned that they had to quit their jobs to take care of family matters during the pandemic. Likewise, many studies have showed that females took on primary household responsibilities when schools were closed and family member were at home, regardless of employment status [51]. Females bear more household responsibilities, which makes them more vulnerable to economic shocks than males [48]. In this regard, our study participants reported that unstable financial situations made them unable to afford health care and medical treatment. These financial burdens directly and indirectly affected their recovery process.

The COVID-19 pandemic has also changed people’s social relationships and the way they communicate with each other. According to Algeri et al.’s study [52], the lockdown was characterized by changes in daily lifestyle (e.g., increased time spent at home, reduced social distance through digital device use). Some studies also suggested that elevated levels of social isolation and being at home lead to negative emotions [53,54,55,56]. In our study, some participants stated that increased time spent at home and changes in time spent with family members made them feel irritable and stressed, which affected their relationships with family members. In addition, some participants indicated that persistent COVID-19 symptoms affected their social relationships directly or indirectly, in terms of social isolation and changes in communication styles. As indicated in the literature, individuals experiencing high levels of social isolation may be negatively treated by the public (e.g., reduced interaction) [53,57,58]. Moreover, as the level of social isolation increases, the level of social support and social connection decreases [59,60]. In our study, participants who shared experiences of social isolation mentioned that many people do not understand the persistent symptoms of long haulers, and they also have different attitudes towards COVID-19 vaccination. This lack of understanding and these disagreements could lead to limitations in the development and maintenance of social relationships. Similarly, Viswanath et al. [61] showed that people’s opinions about vaccination are related to their knowledge of acute and persistent COVID-19 symptoms. Furthermore, participants emphasized that only those who had similar experiences could understand them. The literature also showed that since research on long COVID is still in the beginning stages, the related social implications have not yet been fully elucidated [62]. Not everyone understands the barriers and challenges faced by long haulers, which leaves long haulers without timely and on-point help when they need advice and support the most.

Although social isolation had an impact on the maintenance and development of social relationships among long haulers, this study found that social media provides a novel way for long haulers to communicate, strongly reduce the negative effects of social isolation. The benefits of social media use [63,64] and the power of online platforms in influencing people’s experiences of COVID-19 [65,66] are also demonstrated by other studies. Social isolation and negative social responses to persistent symptoms were the main factors in encouraging long haulers to join peer support groups via online platforms and social media to communicate and share their experiences and gain social acceptance and receive COVID-19-related information. Given that the pandemic has not yet ended, we need to focus on the needs of long haulers regarding social support from their peers and be mindful of online intervention delivery.

This study highlights conflicts in social roles, particularly between work and family roles, as one of the prominent issues experienced by female long haulers. Work–family role conflict occurs when pressures from work and family roles cannot be adjusted in an appropriate way [67]. Thus, working people who could not balance their time and efforts between family and work have suffered from work–family conflict [68]. Similar to our results, previous studies have shown that work–family conflict has a significant impact on the performance of female employees and causes them distress and fatigue [68]. During the COVID-19 pandemic, females have taken on more domestic and caregiving responsibilities [69], culminating triggering multiple role conflicts [69,70]. As reported in our study, females experience job changes during COVID-19. These job changes put them in a difficult situation and led to greater distress [71] as they had to choose between their health and earning a salary to pay for their living expenses [69].

Our findings suggest that employer attitudes during the pandemic could be a key factor influencing role conflict. In a study by Senerat et al. [72], participants experienced anxiety, stress, lack of companionship, fatigue, decreased productivity, and feeling insecure due to the conflicts in the work role. In addition, similar to our findings indicating ignoration by the employers, Kong and Belkin [73] discussed that employees feel neglected by their employers as a common experience of the pandemic. Considering the challenging situations of individuals in the pandemic, they anticipate attention and care from their employers [74]. They expected their employers to provide them with a place where they could feel seen and heard [75]. Schieman et al. [76] described the conflict between family and work roles in COVID-19 as a competition between time, effort, and attention to personal life needs. Several studies have emphasized that balancing work and family responsibilities can help minimize conflict, especially when many people struggle to do well at work while also caring for their family members [75], such as working mothers, who face more significant inter-role conflict [77]. Thus, as mentioned by the participants, support from employers and colleagues in the workplace may help female employees cope better with difficulties related to role conflict at work [76,78,79] and may assist them to handle family and work responsibilities more effectively [80,81].

According to our findings, coronavirus-associated social stigma adversely affects long haulers’ social life. The term stigma refers to the characteristics of individuals that devalue society and consider them unfit for inclusion in mainstream society [82]. According to World Health Organization (WHO), social stigma in the context of health refers to the negative association between individuals or groups with certain characteristics related to specific diseases [83]. According to Bhanot et al. [84], social stigma causes psychological distress by affecting social relationships. Moreover, Imran et al. [85] showed that COVID-19-related social stigma results in loss of trust or respect, rejection, and stigmatizing behaviors. In our study, participants acknowledged that being labeled, treated differently, or stigmatized had a huge impact on their social life. Likewise, other studies have shown that people feel a deep sense of exclusion due to stigmatization [86], which may cause mental disorders [87]. Furthermore, social stigma affects females’ health by being a prominent barrier to improving the diagnostic and treatment procedures for mental health problems (e.g., anxiety and depression) [15]. As in another study evaluating perceived stigma among patients recovering from COVID-19, those who scored higher on post-traumatic stress disorder, anxiety, and depression reported more fatigue and stigma [88]. In our study, participants also reported that internalized stigma, self-blame, and fear of being judged by others exacerbated their psychological and physical burdens. This finding supports previous studies that people suffering from COVID-19 symptoms fear being judged by others [89], delay seeking health care, and being isolated [90]. Likewise, in the Severe Acute Respiratory Syndrome (SARS) outbreak, fear of stigma and being judged by others affected patients’ social behaviors and relationships [91]. Social stigma can undermine social cohesion and lead to social isolation of groups, which may potentially cause more severe health problems and uncontrollable disease outbreaks [83].

## 5. Limitations and Future Directions

Our study is limited by its generalizability. We did not collect information on a number of demographic variables in our sample, which limits our analysis regarding the role of sociodemographic variables on female long haulers’ social life. Moreover, this study is part of an online health intervention among long haulers and recruited potential participants from Facebook groups, so they are a group interested in online intervention and have access to social media. Therefore, the participants may not be representative of the general population. Furthermore, considering the geographical concentration of our participants in eastern states, the results may be limited by selection bias and not representative of long haulers in all states. Like all other qualitative research, our study may also be influenced by researchers’ subjective bias during the process of discussion guide development, transcription coding, and results interpretation. Finally, although two researchers conducted the coding independently, we did not calculate the intercoder agreement because they resolved all disagreements in the final coding through discussion.

Despite these limitations, our results demonstrate how persistent COVID symptoms affect female long haulers’ social life and indicate the importance of changes in various aspects of social life for female long haulers when they face long COVID. This study informs potential interventions to reduce the problems of female long haulers by focusing on physical enhancement, economic empowerment, deepening social relationships, and addressing conflicts in social roles. For example, while healthcare professionals can concentrate on improving the limited physical activities of female long haulers, social workers can help resolve the conflicts in their social roles. Likewise, family and friends can help female long haulers to promote their diminished social relationships, and the government can provide new policies and programs to reduce the financial burdens of these women.

## 6. Conclusions

This study provides insights into the impact of long COVID on the social life of female long haulers. Persistent COVID-19 symptoms not only change the physical health of female long haulers but also lead to conflicts between their social roles. Our findings highlight the need for tailored health promotion strategies and social support for long haulers by considering their altered social life. This study informs the health policies and interventions aimed to facilitate long haulers’ return to normal social life. Future research needs to examine the impact of long COVID on the social life of other groups (e.g., men, people with disabilities, the elderly, and immigrants), who may experience other forms of social role conflict. Furthermore, studying coping strategies among female long haulers and developing innovative programs that include online communication as a health promotion strategy would be beneficial for female long haulers.

## Figures and Tables

**Table 1:** Interview guide.

Topic	Question
Background of COVID-19 infection and its physical related symptoms	When were you diagnosed with COVID-19? What symptoms have you experienced since the infection? Do you experience any long-term (or persistent) symptoms, which last for weeks or even months after recovery? Do these symptoms affect your life? (Social, working, daily life, etc.) What are the biggest challenges that the COVID-19 infection and long-COVID symptoms brought to you?
Psychosocial influences of COVID-19	Did the symptoms affect your moods? Your mental health? (Describe some examples) Do other people know that you have these symptoms? How do they respond it? (Describe specific examples and/or situations)
Social sources for support and resilience	Who do you turn to when you experience stress and distress? How did they help? Do you need any additional resources or help to enhance your physical and mental health? If so, what are they? Are there any things you do to help to overcome bad feelings and bounce up?
Others	Do you have any questions for me about the study and intervention? Is there anything else would you like to share with me?

**Table 2:** Demographic characteristics of female long haulers.

Variable	*n* (Total = 15)	Percent
Age		
20–35	2	13.33
36–50	6	40.00
51–65	6	40.00
>65	1	6.67
Occupation		
Healthcare Provider	5	33.33
Educator	4	26.67
Business Owner	4	26.67
Student	1	6.67
Retired	1	6.67
Living situation		
Live with others	13	86.67
Live alone	2	13.33
Location		
East	10	66.67
Central	3	20
West	2	13.33

## Data Availability

The datasets generated during and analyzed during the current study are not publicly available due to the signed consent agreements around data sharing but are available from the corresponding author on reasonable request.
